# Metabolomics-Microbiome Crosstalk in the Breast Cancer Microenvironment

**DOI:** 10.3390/metabo11110758

**Published:** 2021-11-04

**Authors:** Mysoon M. Al-Ansari, Reem H. AlMalki, Lina A. Dahabiyeh, Anas M. Abdel Rahman

**Affiliations:** 1 Department of Botany and Microbiology, College of Science, King Saud University, Riyadh 11451, Saudi Arabia; myalansari@ksu.edu.sa (M.M.A.-A.); 439203044@student.ksu.edu.sa (R.H.A.); 2 Department of Molecular Oncology, Cancer Biology & Experimental Therapeutics Section, King Faisal Specialist Hospital and Research Centre (KFSHRC), Riyadh 11211, Saudi Arabia; 3 Department of Pharmaceutical Sciences, School of Pharmacy, The University of Jordan, Amman 11942, Jordan; l.dahabiyeh@ju.edu.jo; 4 Metabolomics Section, Department of Clinical Genomics, Center for Genomics Medicine, King Faisal Specialist Hospital and Research Centre (KFSHRC), Zahrawi Street, Al Maather, Riyadh 11211, Saudi Arabia; 5 Department of Biochemistry and Molecular Medicine, College of Medicine, Al Faisal University, Riyadh 11533, Saudi Arabia

**Keywords:** metabolomics, microbiome, microenvironment, breast cancer

## Abstract

Breast cancer, the most frequent cancer diagnosed among females, is associated with a high mortality rate worldwide. Alterations in the microbiota have been linked with breast cancer development, suggesting the possibility of discovering disease biomarkers. Metabolomics has emerged as an advanced promising analytical approach for profiling metabolic features associated with breast cancer subtypes, disease progression, and response to treatment. The microenvironment compromises non-cancerous cells such as fibroblasts and influences cancer progression with apparent phenotypes. This review discusses the role of metabolomics in studying metabolic dysregulation in breast cancer caused by the effect of the tumor microenvironment on multiple cells such as immune cells, fibroblasts, adipocytes, etc. Breast tumor cells have a unique metabolic profile through the elevation of glycolysis and the tricarboxylic acid cycle metabolism. This metabolic profile is highly sensitive to microbiota activity in the breast tissue microenvironment. Metabolomics shows great potential as a tool for monitoring metabolic dysregulation in tissue and associating the findings with microbiome expression.

## 1. Introduction

Breast cancer is a heterogeneous disease and one of the world’s most prevalent malignancies. According to the World Health Organization (WHO), 2.3 million women were diagnosed with breast cancer in 2020, with 685,000 deaths globally (https://www.who.int/news-room/fact-sheets/detail/breast-cancer, accessed on 15 September 2021). Lately, Breast cancer incidence has increased to 29.7% among Saudi women [1]. Several risk factors are associated with breast cancer; they are mainly classified as modifiable and non-modifiable factors. Non-modifiable risk factors include age, menopause, family history, hormonal variations, and genetic susceptibility. Modifiable risk factors such as diet, lack of physical activity, obesity, alcohol consumption, and oral contraceptive can be changed if appropriate measures are taken [1,2,3]. Normally, patients develop breast-related signs such as lumps, size alteration, pains, and nipple fluid discharge [4]. Although women are at a higher risk of developing breast cancer than men, breast cancer may occur in males, who represent less than 1% of overall breast cancer cases [5]. Despite the low incidence of breast cancer in males, the mortality rate is considered high as the disease is often only discovered at the final stage. Compared to females, breast tumors in males are more often of the ductal carcinoma type and estrogen- and progesterone-receptor positive [6].

There are five main molecular subtypes of breast cancer that are associated with the expression of three receptors in tumor cells, namely estrogen (ER), progesterone (PR), and human epidermal growth factor receptor-2 (HER2); Luminal A cancers largely correspond to ER or PR positive, HER2 negative, and low histological grade/proliferation rate, while Luminal B tumors display relatively lower levels of ER or PR expression, and either exhibit HER2 amplification, high histological grade/high proliferation, or both. The HER2-enriched group (ERBB2) consists of ER-negative tumors and expresses genes mapping to the HER2 amplicon. Additionally, triple-negative breast cancer phenotype (TNBC) is formed by basal-like cancers characterized by low or /lacking levels of expression of ER and ER-related genes (including PR) and the frequent absence of HER2 overexpression. Normal breast-like subtype tumors show remarkable similarities with normal breast and fibroadenomas samples at the messenger Ribonucleic Acid (mRNA) expression level. There are three histological grades: grade 1—well-differentiated; grade 2—moderately differentiated; and grade 3—poorly differentiated [7,8,9].

Tumor microenvironment cells (TME) play a crucial role in cancer development and progression [10]. The heterogeneity of the TME mainly consists of the extracellular matrix (ECM) and various types of tumor stromal cells, including immune and inflammatory cells, endothelial cells, adipocytes, bone marrow-derived cells, and fibroblasts [11]. Endothelial cells are critical to the development of tumor angiogenesis, which provides metastatic tumor cells entry to the circulatory system [12]. Fibroblast cells are considered one of the most abundant and significant types of cells in the TME. Normally, fibroblasts play a key role in wound healing, epithelial differentiation regulation, and inflammation [13,14]. However, they are present in either activate or inactivated forms inside tumors, commonly known as cancer-associated fibroblast (CAF) or/myofibroblasts [13]. In cancer, CAFs trigger invasion, progression, and metastasis [15].

Before, the breast was initially thought to be sterile. Nowadays, multiple studies confirm that resident microbes in the breast [16] can be considered one component of the TME [17]. Therefore, the TME may provide favorable conditions for these microbes to survive and evolve [16]. Therefore, microbiota play a critical role in immune system development by promoting inflammation or suppressing anti-tumor immunity (as reviewed extensively in [18,19]). Thus, the dysbiosis of microbiota can contribute to breast cancer progression and other health conditions. The crosstalk between other types of microenvironment components, especially fibroblasts and the microbiome, as well as the microbiome more generally, is understudied.

Recently, researchers has shown great interest in understanding and connecting the inflammation mechanism involved in breast cancer with the breast tissue microbiome [2,20,21]. Disturbance of the microbiome has been linked to chronic diseases and malignancies, including breast cancer. Microbial alterations observed in breast cancer highlight the possible role of microbiota in breast cancer development, prevention, and management [3]. Microbiome expression is associated with the excreted metabolome, which helps study the disease phenotypes and develop biomarkers for disease management. This review introduces updated literature on the connection between the TME and breast cancer development, and discusses the association between the tissue microbiome and metabolic changes in disease development.

## 2. Microbiome: An Overview

The human microbiome is defined as the full array of the diverse microorganisms (microbiota) that live on and in humans, as well as their genetic materials. It is considered one of the leading environmental factors in disease development [20,21], with *Firmicutes*, *Bacteroidetes*, *Proteobacteria*, and *Actinobacteria* the dominant species [20,21]. Human microbiota manifestation is influenced by multiple environmental and physiological changes, including age, sex, race, geography, diet [22,23], host genetics and lifestyle, drugs like antibiotics [24,25], and interaction with the immune system [26] and metabolic pathway [27]. Several studies have linked the human microbiome with important health and disease conditions [28]. For instance, the gut microbiome was not significantly altered in premenopausal breast cancer patients compared to control. However, exceeded number of *Escherichia coli*, *Citrobacter koseri*, *Acinetobacter radioresistens*, *Enterococcus gallinarum*, *Shewanella putrefaciens*, *Erwinia amylovora*, and *Actinomyces* spp. was significantly reported in the postmenopausal group. However, the abundance of *HPA0247*, *Salmonella enterica*, *Fusobacterium nucleatum*, *Eubacterium eligens*, and *Roseburia inulinivorans* was lower in postmenopausal groups [29] (as reviewed extensively in [30,31,32,33]). A urinary-based microbiome study conducted in female breast cancer patients showed a high number of *Corynebacterium*, *Staphylococcus*, *Actinomyces*, and *Propionibacteriaceae*, and diminished number of *Lactobacillus* [34]. A previous study on the human oral microbiome using 16S rRNA Pyrosequencing and microarray reported abundant *Firmicutes*, *Proteobacteria*, *Bacteroidetes*, *Actinobacteria*, and *Fusobacteria* phyla. In addition, *Streptococcus*, *Veillonella*, *Leptotrichia*, *Prevotella*, and *Haemophilus* were common genera [35]. Herein, this review focuses on the breast tissue microbiome associated with breast cancer progression [32].

## 3. Methods for Studying the Microbiota

**Figure 1:**
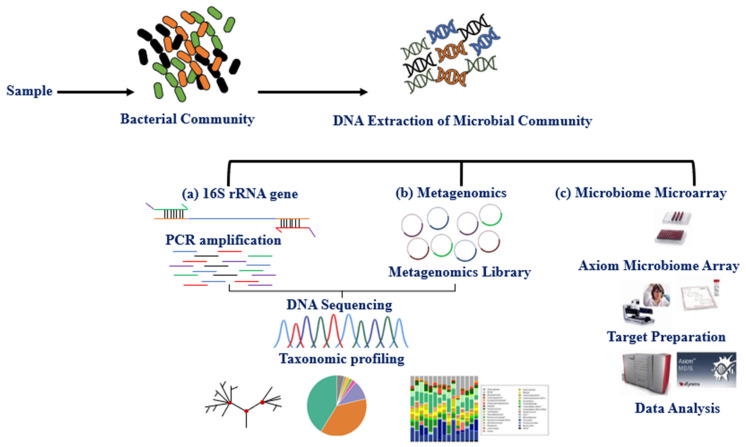
Workflow of common methods for studying the breast tissue microbiome. After extracting microbial DNA from breast tissue or cell lines. (**a**) a PCR amplification based on the 16S rRNA gene of variable regions (V1-V9) is conducted using specific primers to bacterial sequence species, where 16S rRNA is a shared region between bacteria and archaea. (**b**) Metagenomics is based on whole DNA sequencing generated from the sample, and the reads are matched with the library specific to the particular species. 16S rRNA gene provides phylogeny and community composition. Metagenomics also provides the community composition and function of genes. (**c**) Microbiome Microarray is designed using the high-density Axiom platform for microbiome analysis, containing ~1.38 million DNA probes specific to microbiota species.

The microbiota can be studied directly using traditional culture-dependent or molecular approaches and indirectly through its association with other biomolecules or omics approaches such as epigenetics and metabolomics. The primary molecular technique for studying microbiota expression is DNA amplification of hypervariable regions using polymerase chain reaction (PCR). Microbiota identification (sequencing) and expression level are obtained using next-generation sequencing technologies (NGS) and microarray [35]. Multiple studies have explored the variable regions (V1–V9) 16S rRNA, shared by bacteria and archaea, using the NGS, whole-genome shotgun sequencing (WGS), and DNA microarray (e.g., PathoChip) techniques [36,37,38]. These techniques together have contributed to the study of the human microbiome and established an association between imbalance in the microbiome (dysbiosis) and disease phenotypes [39,40,41,42]. The International Human Microbiome Standard (www.microbiome-standards.org, accessed on 25 August 2021) and the Microbiome Quality Control project (www.mbqc.org, accessed on 25 August 2021), have developed standard operating procedures (SOP) designed to improve data quality and comparability in the human microbiome field (Figure 1) [38].

## 4. Breast Tissue Microbiome

The microbiota’s dysbiosis has contributed significantly to breast cancer progression, and other health conditions, as reviewed elsewhere [21,24]. Microbes may, directly or indirectly, influence the development of breast cancer. The direct effect involves microbes on skin/breast tissue that contribute to breast cancer progression via contact with breast tissue. On the other hand, the indirect effect involves structural and functional components of bacteria, secretion products (e.g., quorum sensing peptides), or bacterial metabolites [21,24,34,43]. Several studies that describe the correlation between tissue microbiome dysbiosis and breast cancer development have revealed distinct species expression in patients compared to healthy individuals (Table 1) [44,45]. Urbaniak et al. [20] reported a higher abundance of *Prevotella*, *Lactococcus*, *Streptococcus*, *Corynebacterium*, and *Micrococcus* in healthy women, while *Bacillus*, *Staphylococcus*, *Enterobacteriaceae*, *Comamondaceae*, and *Bacteroidetes* were more abundant in women with breast cancer. In a more comprehensive study based on tissue samples collected from The Cancer Genome Atlas (TCGA), *Mycobacterium fortuitum*, and *Mycobacterium phlei* were found abundant in breast cancer tissues (*n* = 668) compared to the normal adjacent tissues (*n* = 72) [46]. Another study offered substantial evidence connecting breast cancer development to microbiome diversity and expression, where *Methylobacterium* growth was significantly decreased in cancer patient breast tissues [34]. A previous study reported a relative increase of *Methylobacterium radiotolerans* in tumor tissue versus *Sphingomonas yanoikuyae* in healthy adjacent tissue. The bacterial DNA load showed an inverse correlation with the stage of breast cancer disease [2,47]. Therefore, bacterial load may be linked to reduced gene expression of the antibacterial response gene in advanced-stage breast cancer [2]. Costantini et al. [45] studied the multi-hypervariable region of the 16S-rRNA gene and found that the V3 region is the most informative for breast tissue microbiota. The microbiota imbalance may lead to downstream malfunction of the immune system, permitting tumor development [19]. Of note, most of these studies have sequenced the 16S rRNA gene using qPCR, NGS, or DNA microarray (PathoChip) methods for bacterial identification, as summarized in Table 1.

## 5. Metabolomics for Studying Breast Cancer

Metabolomics is the global identification and quantification of a set of small molecules such as carbohydrates, nucleic acids, amino acids, and lipids within a biological system. A promising emerging analytical approach assesses the global metabolic expression associated with a certain health condition. Several biological materials might be used in this approach, including tissue samples. Metabolomics relies on the use of advanced analytical instruments with bioinformatics. Depending on the analytical platform, metabolomics might be studied in a targeted or untargeted fashion using nuclear magnetic resonance (NMR) or mass spectrometry (MS) [50]. Typically, MS is coupled with separation techniques such as liquid and gas chromatography (LC and GC, respectively) to improve metabolome coverage [51]. The latter is affected by study confounders (e.g., age, gender, ethnicity, diet, disease state, and drug exposure) that need to be addressed before exploring the disease-associated metabolic panel [52,53,54]. Over the years, metabolomics studies have received increasing interest as a promising advanced analytical approach for biomarker discovery, disease mechanism exploration, and therapeutic target identification [55]. In terms of cancer, metabolomics has demonstrated exceptional potential to provide a perspective on interactions between the tumor, host, and environment and within the cancer itself [56]. Metabolomics has provided new insights into different biological and clinical aspects of tumors, including profiling metabolic abnormalities associated with specific cancer types and monitoring cancer progression and therapeutic liabilities [57]. In breast cancer, most metabolomics studies use tumor tissues or cell lines with the goals of providing distinctive profiles for each subtype, distinguishing cancer or cancer with metastasis from normal tissues, predicting biomarkers for disease pathophysiology, monitoring treatment, and identifying new therapeutic targets [58].

The TME highly affects the metabolism of cancer cells. Breast cancer cells show characteristic pathological metabolic changes due to a complex rearrangement of the cellular energy signaling pathways. The metabolic pathways highly affected in cancer are glycolysis and mitochondrial oxidation (tricarboxylic acid cycle, TCA cycle) [33]. Disturbances to amino acid metabolism (mainly glutamine metabolism), nucleotide and lipid and fatty acid pathways, and protein translation have also been reported [52,59,60,61,62]. Changes in metabolism have a significant role in supporting the proliferation and angiogenesis of breast cancer cells. Aerobic glycolysis, or the Warburg effect, is a hallmark phenomenon in cancer. Most of the energy production comes through glycolysis, as opposed to mitochondrial oxidative phosphorylation in normal differentiating cells, in order to support the high proliferative activity of cancer cells [63]. A study by Martinez-Outschoorn et al. [64] on human cell lines demonstrated that loss of function in BRCA1 mutation led to the production of hydrogen peroxide and oxidative stress in epithelial breast cancer cells and in the stromal fibroblast microenvironment, and resulted in an increased expression of monocarboxylate transporter 4 (MCT4) to shuttle L-lactate out of cells. This finding highlighted the potential role of antioxidant therapies in breast cancer prevention. Moreover, the same study reported a loss of caveolin-1 (a marker for breast cancer progression) in CAFs, attributed to mutations of BRCA1 as a result of high glycolysis in stromal cells [64]. Lactate dehydrogenase (LDH) is one of the main TME metabolic enzymes, and is essential for converting pyruvate to lactate during glycolysis. Four LDH genes are known in the vertebrates, i.e., LDHA, LDHB, LDHC, and LDHD, which are critically involved in cancer metabolism [65]. LDHA and LDHB, contribute to tumor stroma metabolic interaction and metabolic fuel exchange, and hence could serve as anticancer therapeutic targets. In particular, LDHB expression may serve as a predictive metabolic marker for therapeutic response in various cancers. In breast cancers, the expression of the LDHB gene (encodes LDH-1) has been used to evaluate response to neoadjuvant chemotherapy [66]. A previous study conducted on patients with basal-like cancers reported high expression levels of LDHB [67]. 

On the other hand, loss of LDHB expression in breast cancer (adenocarcinoma) tissues and cell lines due to promoter hypermethylation has been linked to metastatic development [68]. Various studies have shown that fibroblasts play a crucial role in developing tumor cells in the TME. Glycolytic tumor cells can generate lactate by converting glucose to pyruvate and generating the NAD+ needed for continuing glycolysis, an alternative route to the oxidative phosphorylation that occurs in normal cells. The increased concentration of lactate in the TME triggers MCT1, LDHB expression in the nearby stromal cells such as hMSCs/CAFs [69].

Metabolomics represents a promising advanced analytical approach for investigating the metabolome of breast cancer tissue. Metabolomics has identified specific metabolic alterations between four intrinsic subtypes of breast cancer (luminal A and B, HER2-enriched (ER−, HER2+), and TNBC) [33], and between ER+ and ER− breast cancer tissue metabolomes [55]. Moreover, using multiple tracer stable isotope resolved metabolomics, Lane et al. [70] have investigated the functional differences between different breast cell types (one primary breast and three breast cancer cells) and mouse tumor xenografts. They found that pyruvate carboxylation was activated in breast cancer versus primary cells and reported significant differences in glucose metabolism between in vivo and in vitro conditions emphasizing the influence of 3D cell architecture and/or tumor microenvironment [70]. Additionally, metabolomic studies have successfully enabled the identification of potential biomarkers for discriminating between breast cancer and normal tissues [60,62,71,72,73].

Breast cancer preferentially grows in adipocyte-enriched environments, which can induce changes in the composition of cells surrounding the tumor microenvironment affecting tumor cell proliferation [74]. Several lipidomics studies have highlighted lipid metabolism in breast cancer tissues compared to normal breast tissue [75,76,77,78]. A summary of the findings of the above metabolomics and lipidomics studies is presented in Table 2.

## 6. Interaction between Microbiome and Metabolomics in Breast Cancer 

Recently, interest in studying the association between the microbiome and metabolic alteration in cancer has increased. Microbiome metabolites can be critical modulators of the TME by regulating, either positively or negatively, vital processes such as inflammation, proliferation, and cell death [79]. However, research investigating the interaction between the microbiome and the metabolome in breast cancer is limited. Only a few reports have highlighted the association between the breast microbiome and metabolome in the breast cancer microenvironment. A previous study reported a higher abundance of *Bacillus cereus* in breast cancer patients compared with healthy controls. *Bacillus cereus* metabolizes progesterone into 5-alpha-pregnane-3,20-dione, stimulating cell proliferation and tumor progression [20,80]. Moreover, dysbiosis of the gut microbiome leads to elevated activities of β-glucuronidase, which is responsible for estrogen reactivation through the deconjugation of conjugated estrogens, and hence, an increased risk of estrogen-related conditions such as breast cancer [81,82]. A recent LC-MS metabolomics study reported a correlation between the gut microbiome and choline metabolism in breast cancer patients. The lower abundance of *Faecalibacterium* was linked to the upregulation of phosphocholine levels. [83]. The study suggested that combining flora-metabolites with the flora-bacteria (e.g., *Faecalibacterium* combined with phosphocholine) might serve as promising diagnostic biomarkers for breast cancer, and that *Faecalibacterium* may suppress breast cancer proliferation and invasion by inhibiting IL-6 signal transducers and activators of the transcription 3 (STAT3) pathway [83]. Lithocholic acid is a bacterial metabolite that could influence cancer cell proliferation through activation of the Takeda G-protein-coupled receptor 5 (TGR5) [84]. Bacterial metabolites, lithocholic acid, short-chain fatty acids, indole-propionic acid (IPA), or cadaverine can limit the proliferation of breast cancer cells [84,85,86]. These findings suggest that a deeper understanding of the link between microbiome and metabolome in breast cancer may provide new biomarkers as well as, therapeutic and prevention strategies.

## 7. Conclusions

Disturbances in the microbiota contribute to several pathological conditions, including breast cancer. The presented work contains recent studies on the impact of the breast tissue microbiome and metabolic alteration on the breast cancer microenvironment. Despite the increasing interest in the link between the microbiome and the metabolome in breast cancer, studies investigating the association with breast cancer are still very limited. The use of metabolomics, a sophisticated analytical approach, will precisely demonstrate the link between microbiome and metabolome in the microenvironment in breast cancer in order to aid in diagnosis, prediction, and treatment response. Notably, sample preparation in metabolomic studies using cells or tissues can highly impact the level, stability, and type of metabolites identified. Therefore, appropriate standardized protocols to estimate the metabolic changes in breast cancer cells or tissues remain essential. Future studies on a large-scale, including retrospective and prospective studies, are needed to provide new insights into the implications of the microbiome and metabolomics in the breast cancer microenvironment and how their complex interactions affect the tumor microenvironment’s molecular networks and pathways. Additionally, in-depth studies investigating the role of the gut microbiome in breast cancer metabolism and its link to the breast cancer microbiome remain an urgent subject for further investigation.

## Figures and Tables

**Table 1:** Summary of studies investigating the alteration of breast tissue microbiome in breast cancer.

Sample Type and Size				Method	Variable Region	Changes to the Microbiome			Ref.
Healthy	Benign	Cancer	Adjacent			Healthy Patients	Cancer Patients	Adjacent	
		20	20	NGS	V4		↑ *Methylobacterium radiotolerans*	↑ *Sphingomonas yanoikuyae*	[2]
24		17	22	NGS	V3–V4		↓ *Methylobacterium*		[34]
23	13	45		NGS	V6	↑ *Prevotella*, *Lactococcus*, *Streptococcus*, *Corynebacterium*, and *Micrococcus*	↑ *Bacillus*, *Staphylococcus*, *Enterobacteriaceae*, *Comamondaceae*, and *Bacteroidetes.*		[20]
		668	72	NGS	V3–V5		↑ *Mycobacterium fortuitum* and *Mycobacterium phlei*		[46]
5, Canadians	11	27		NGS	V6		The most abundant taxa in the Canadian samples were: *Bacillus* (11.4%), *Acinetobacter* (10.0%), *Enterobacteriaceae* (8.3%), *Pseudomonas* (6.5%), *Staphylococcus* (6.5%), *Propionibacterium* (5.8%), *Comamonadaceae* (5.7%), *Gammaproteobacteria* (5.0%), and *Prevotella* (5.0%).		[48]
5, Irish		33					The most abundant taxa in the Irish samples were: *Enterobacteriaceae* (30.8%), *Staphylococcus* (12.7%), *Listeria welshimeri* (12.1%), *Propionibacterium* (10.1%), and *Pseudomonas* (5.3%). ↑ *Escherichia coli*		[48]
20		50, BRER 34, BRHR 24, BRTP 40, BRTN		PathChip array			Unique and common microbial signatures in the major breast cancer types are summarized in Table 1 in (51) All four breast cancer types had dominant signatures for *Proteobacteria* followed by *Firmicutes*. *Actinomyces* signatures were also detected in each breast cancer types.		[49]
		9, CNB 7, SEB 3, Both	9, CNB 7, SEB 3, Both	NGS	V2–V4 V6–V9		*Proteobacteria* are the most abundant phylum followed by *Firmicutes*, *Actinobacteria*, and *Bacteroidetes*. The presence of the genus *Ralstonia* is associated with breast tissue. The relative abundance of *Methylobacterium* was different in certain patients.		[45]

NGS: Next-generation sequencing, qPCR: quantitative Polymerase chain reaction, BRER: endocrine receptor (estrogen or progesterone receptor) positive, BRHR: human epidermal growth factor receptor 2 (HER2) positive, BRTP: triple positive (estrogen, progesterone, and HER2 receptor-positive), BRTN: triple-negative (absence of estrogen, progesterone, and HER2 receptors), CNBs: core needle biopsies, SEBs: surgical excision biopsies. Up and down arrows refer to up- and down-regulated bacteria, respectively.

**Table 2:** Metabolomics in Breast Cancer. This is a summary of multiple studies used metabolomics to study breast cancer in different biological matrices (√).

Biological Materials		Approach (Targeted/Untargeted)	Altered Metabolites and Metabolic Pathways	Ref.
Cell Line	Tissue			
	√	GC–TOFMS (Targeted)	Increased beta-alanine, 2-hydroyglutarate, glutamate, xanthine, and decreased glutamine in ER− subtype compared to ER+ Beta-alanine has shown the most significant change between breast cancer ER− and ER+	[55]
	√	LC-/MRM-MS GC-MS (Targeted and Untargeted)	Up-regulation of histidine, glutamine, tyrosine, creatine, phenylalanine, lactic acid, adonitol, glutamic acid, and downregulation of 3,7-cholest-5-ene. The study identified tryptophan, tyrosine, and creatine, in serum and tissue as potential markers for invasive ductal carcinoma (IDC).	[60]
	√	GC-MS	cytidine-5-monophosphate/pentadecanoic acid metabolic ratio was a significant discriminator between cancer and normal tissues	[62]
√		NMR FT-ICR-MS	glutaminolysis is connected to pyrimidine ring synthesis in all cell types anaplerotic pyruvate carboxylation was activated in breast cancer versus primary cells	[70]
√		LC-MS/MS (Targeted)	Glycine biosynthetic pathway was highly correlated with fast proliferating breast cancer cells	[71]
√	√	LC-MS, GC-MS (Targeted)	Higher level of aspartate in breast cancer tissues than adjacent non-tumor tissues, MCF-7 cell line than in MCF-10A cells	[72]
	√	MALDI MSI (Targeted)	Adenosine diphosphate, adenosine monophosphate, adenosine triphosphate, aspartate, citrate, deoxycytidine diphosphate, fructose 1,6-bisphosphate, glutamate, glutathione, glutathione disulfide, guanosine diphosphate, N-acetylaspartate, NADH, UDP-glucose, DP-N-acetylglucosamine, UDP, UMP	[73]
	√	GC-TOF-MS (UPLC-MS)	Phospholipids, including PtdCho-s, phosphatidylethanolamines, phosphatidylinositol, sphingomyelin, triglycerides	[75,76]
	√	LC-ESI-MS/NMR (Targeted)	Up-regulation of choline, phosphocholine, glycerophosphocholine	[77]
	√	HR MAS MRS (Untargeted)	Up-regulation of phosphocholine, glycine, taurine, creatine, lactate, ascorbate, and downregulation of glucose	[78]

√ indicates type of biological material used in the study.
